# An eQTL variant of *ZXDC* is associated with IFN-γ production following *Mycobacterium tuberculosis* antigen-specific stimulation

**DOI:** 10.1038/s41598-017-13017-8

**Published:** 2017-10-09

**Authors:** Fabienne Jabot-Hanin, Aurélie Cobat, Jacqueline Feinberg, Marianna Orlova, Jonathan Niay, Caroline Deswarte, Christine Poirier, Ioannis Theodorou, Jacinta Bustamante, Stéphanie Boisson-Dupuis, Jean-Laurent Casanova, Alexandre Alcaïs, Eileen G. Hoal, Christophe Delacourt, Erwin Schurr, Laurent Abel

**Affiliations:** 1 Laboratory of Human Genetics of Infectious Diseases, Necker Branch, INSERM U1163 Paris, France; 2 Paris Descartes University, Sorbonne Paris Cité, Imagine Institute, Paris, France; 3 Program in Infectious Diseases and Immunity in Global Health, The Research Institute of the McGill University Health Centre, Montreal, Canada; 4 McGill International TB Centre, McGill University, Montreal, Canada; 5 Department of Human Genetics and Department of Medicine, McGill University, Montreal, Canada; 6 Université Pierre et Marie Curie, UF d’Histocompatibilité et Immunogénétique, Département d’Immunologie, Groupe Hospitalier Pitié Salpêtrière - Charles Foix, Paris, France; 7 Centre de Lutte Anti-Tuberculeuse, Centre Hospitalier Intercommunal de Créteil, Créteil, France; 8 St Giles Laboratory of Human Genetics of Infectious Diseases, Rockefeller Branch, Rockefeller University, New York, NY USA; 9 Molecular Biology and Human Genetics, MRC Centre for Molecular and Cellular Biology, DST/NRF Centre of Excellence for Biomedical TB Research, Faculty of Health Sciences, Stellenbosch University, Tygerberg, South Africa; 10 Pediatric Pneumology Unit, Necker Hospital for Sick Children, AP-HP, Paris, France; 11 Howard Hughes Medical Institute, New York, NY USA; 12 Pediatric Hematology-Immunology Unit, Necker Hospital for Sick Children, AP-HP, Paris, France

## Abstract

There is a large inter-individual variability in the response to *Mycobacterium tuberculosis* infection. In previous linkage analyses, we identified a major locus on chromosome region 8q controlling IFN-γ production after stimulation with live BCG (Bacillus Calmette-Guérin), and a second locus on chromosome region 3q affecting IFN-γ production triggered by the 6-kDa early secretory antigen target (ESAT-6), taking into account the IFN-γ production induced by BCG (IFNγ-ESAT6_BCG_). High-density genotyping and imputation identified ~100,000 variants within each linkage region, which we tested for association with the corresponding IFN-γ phenotype in families from a tuberculosis household contact study in France. Significant associations were replicated in a South African familial sample. The most convincing association observed was that between the IFNγ-ESAT6_BCG_ phenotype and rs9828868 on chromosome 3q (*p* = 9.8 × 10^−6^ in the French sample). This variant made a significant contribution to the linkage signal (*p* < 0.001), and a trend towards the same association was observed in the South African sample. This variant was reported to be an eQTL of the *ZXDC* gene, biologically linked to monocyte IL-12 production through CCL2/MCP1. The identification of rs9828868 as a genetic driver of IFNγ production in response to mycobacterial antigens provides new insights into human anti-tuberculosis immunity.

## Introduction

Tuberculosis remains a major public health concern, with approximately 10.4 million new cases and 1.8 million deaths due to the disease in 2015^1^. While an estimated one third of the world population is estimated to be infected with *Mycobacterium tuberculosis*, only about 10% of infected individuals go on to develop clinical disease^2^. There is no direct proof of latent *M. tuberculosis* infection (hereafter referred to simply as LTBI) in exposed individuals, and the infection phenotype is inferred indirectly from quantitative measurements of antimycobacterial immunity^2^. The tuberculin skin test (TST) is the most widely used method^3^, but additional assays testing for LTBI on the basis of *in vitro* evaluations of T-cell antimycobacterial immunity, have been developed over the last 15 years^4^. These tests measure the production of interferon–gamma (IFN-γ) by circulating leukocytes (IFN-γ release assays, IGRAs) in response to M. tuberculosis antigens, such as the 6 kDa early secretory antigen target (ESAT-6)^5^.

Based on TST and IGRA results, an estimated 10%–20% of subjects do not become infected with *M. tuberculosis* despite sustained exposure and, hence, never develop disease^2,6^. Several studies focusing on TST reactivity have provided evidence for the role of human genetic factors in different steps of the infection process^7–11^. IGRA phenotypes have been less studied, but the heritability of IFN-γ secretion has been estimated at about 43% following BCG stimulation and 58% following ESAT-6 stimulation in South Africa^12^, and at 17%–48% following stimulation with *M. tuberculosis* antigens, including ESAT-6, in Uganda, depending on the TST status of those tested^13,14^.

In a recent linkage analysis, we identified two major loci controlling IFN-γ production induced by mycobacterial stimuli in populations of various ethnic origins living in different *M. tuberculosis* exposure settings^15^. A locus on chromosome 8q12-22 was implicated in IFN-γ production after live BCG stimulation, whereas a second locus on 3q13-22 was found to control IFN-γ levels upon ESAT6 stimulation, accounting for some of the IFN-γ production induced by BCG. In this study, we performed comprehensive fine mapping for these two loci, through high-density genotyping and imputation in the two familial samples used in our previous study^15^.

## Materials and Methods

### Subjects and families

A prospective study of household TB contacts was conducted in the Val-de-Marne, in the Greater Paris region, as previously described^15,16^. Val-de-Marne is an area of low TB endemicity, with an annual TB incidence of 22.1 cases per 100,000 at the time of study, versus an overall incidence of 8.8 per 100,000 in France. From April 2004 to January 2009, household contacts exposed to a patient with culture-confirmed pulmonary TB were enrolled in the context of a general screening procedure (Supplemental Methods). This study was approved by the French Consultative Committee for the Protection of Persons Involved in Biomedical Research (CCPPRB; an IRB) of Henri Mondor Hospital (Créteil, France). Written informed consent was obtained from all study participants, and from the parents of all minors/children enrolled. As a replication cohort, we used a familial sample from the Ravensmead and Uitsig suburbs near Tygerberg, Cape Town, South Africa, where TB is hyperendemic^17^. The Tygerberg families were part of the sample used to map the *TST1* and *TST2* loci^10^, and to study the heritability of antimycobacterial immunity^12^.

We confirm that all the methods used were performed in accordance with relevant guidelines and regulations.

### Measurement of IFN-γ production

For the Val–de-Marne sample, blood samples were collected from each individual and peripheral blood mononuclear cells (PBMCs) were isolated and activated with ESAT-6, PPD, live BCG, and phytohemagglutinin (PHA), as previously described^15^. For the Cape Town sample, IGRAs were performed in quadruplicate on whole blood, with BCG, PPD, ESAT-6, and PHA used for stimulation, as described in our previous study^18^. IFN-γ levels were determined 3 and 7 days after stimulation, but, to ensure comparability with the French discovery sample, we restricted the analysis to the measurements made on day three, as previously discussed^15^.

### Phenotypes and covariates of interest

We used the same phenotypes and covariates as for the linkage analyses^15^, and we focused on the two phenotypes for which significant evidence for linkage was obtained. The first phenotype corresponds to IFN-γ production following BCG stimulation after classical log-transformation and subtraction of the non-stimulated control value. This phenotype, IFNγ-BCG, was adjusted by linear regression for age and covariates relating to individual levels of exposure to *M. tuberculosis*, as previously described^15^. The second phenotype, IFNγ-ESAT6_BCG_, corresponds to IFN-γ production after ESAT-6 stimulation, which was assessed with the same strategy as for the first phenotype. It was further adjusted for IFNγ-BCG, to isolate the more specific response to the ESAT-6 antigen, taking into account the overlapping effects of BCG and ESAT-6 stimulation. The distributions of the two adjusted phenotypes were close to normality (Figure S1).

### Genotyping and Imputation

For the French sample, we used the Illumina HumanOmniExpressExome BeadChip to genotype children and their parents for the genetic association analysis. Individuals with a call rate <90% and duplicates based on identity-by-descent statistics calculated with PLINK1.9 software^19,20^ were removed from the analysis. Single-nucleotide polymorphisms (SNPs) with a call rate <99% were also removed from the analysis. Following quality control filtering, 743,735 high-quality autosomal SNPs and 489 individuals (out of the 528 individuals previously used in the linkage analyses^15^) from 232 families for whom phenotyping data were available were retained for the analyses. The Tygerberg sample was genotyped with the Illumina HumanOmni2.5 BeadChip and, after quality control according to the same criteria as for the French sample, we retained a total of 2,241,954 autosomal SNPs from 373 individuals from 157 families for whom phenotyping data were available, for association analyses.

From the genotyped SNPs, we imputed additional SNPs across the two linkage regions on chromosomes 3 and 8, using the 1000 Genomes Phase 1 reference panel to increase the density of markers in these regions. The two regions of interest extended from 115 Mb to 139 Mb on chromosome 3, and from 61 Mb to 91.5 Mb on chromosome 8, as defined in our previous study^15^. Indeed, given the variability of estimates of location in linkage studies of complex traits, and the slight differences in phenotype definition between the samples used to determine the linkage loci position, it seems reasonable to consider a rather large confidence interval based on the summed LOD scores curves obtained for Val-de-Marne and Tygerberg sample, in our original linkage analyses^15^. For imputation, we first used SHAPEIT software^21^ to pre-phase separately the Illumina HumanOmniExpressExome and HumanOmni2.5 M genotype data for SNPs that passed quality control. We then used IMPUTE2^22,23^ with the 1000 Genomes Phase 1 integrated reference panel to impute the SNP genotypes for the two samples. Imputed SNPs with an information criterion >0.6 and a minor allele frequency (MAF) >0.02 were retained for further analyses. Imputed SNPs significantly associated with either of the two phenotypes of interest were genotyped in the two samples with the high-throughput SEQUENOM iPLEX MassARRAY platform or TaqMan SNP genotyping assays (Applied Biosystems Inc., Foster City, CA).

### Association analysis

Linkage signals were mostly and primarily identified in the Val-de-Marne sample and replicated in the Tygerberg sample. We therefore also used a two-step strategy for association analysis. We first performed a region-wide association analysis on the larger Val-de-Marne sample, and we then tested the replication of the most significant signals in the two regions of interest in the Tygerberg sample. This strategy was also driven by the fact that phenotypes were very similar in the two samples, but not identical, precluding a combined analysis.

Analyses of association between the high-quality SNPs and the two phenotypes (IFNγ-BCG and IFNγ-ESAT6_BCG_) were performed with linear mixed models (LMM) in GEMMA software^24^ to take into account the familial relationships within our samples. The LMM approach is appropriate and robust for family-based association studies, and generally provides a higher power than traditional family-based methods^25^. The relationship matrix used in the regression model was estimated with genotyped genome-wide SNP data and the imputed dosage data were used first-line in the association analyses. In addition, we also performed a principal component analysis (PCA) for the French sample, with the EIGENSTRAT method^26^ as previously described^15^. The five first principal components were used as fixed covariates for adjustment in the association analyses for the Val-de-Marne cohort, to take into account the ethnic heterogeneity of the cohort in LMM analyses, as previously described^27^. Based on this PCA, we classified the individuals of the French sample into three subpopulations: Caucasian individuals (grouping together all individuals of European or North African origin), individuals originating from sub-Saharan African and those of Asian origin.

Each of the two regions of interest has a length corresponding to about 1% of the whole genome. We therefore considered 5 × 10^−6^ to be a reasonable region-wide significance threshold in our analyses, based on a genome-wide threshold of 5 × 10^−8^. We checked that this threshold was appropriate by estimating more accurate region-wide thresholds based on the effective number of independent markers in each region^28^, and by taking into account the observed genomic inflation factors for each phenotype (Supplemental Methods).

SNPs yielding a p-value for association < 5 × 10^−5^ in the Val-de-Marne sample with at least one of the inheritance models tested (additive, recessive or dominant) were assessed in replication analyses in the Tygerberg sample, in which we checked for associations in the same direction for the selected alleles. We excluded the imputed SNPs missing from the 1000 Genomes project Phase 3^29^ from the replication analysis, to focus on the most reliable variants. Following the replication analysis, we selected SNPs for genotyping if they were associated with a *p*-value < 0.05 (one-tailed) in the Tygerberg sample or if they had an initial *p*-value < 10^−5^ in the Val-de-Marne sample and a trend for association was observed in the Tygerberg sample (i.e. a one-tailed p-value <0.5 with the same genetic model as for the French sample). A final association analysis was conducted on the genotyped SNPs in the two samples, with the same genetic model.

### LOD score contributions

We investigated whether the associated variants could, at least to some extent, explain the two linkage signals, by adjusting the corresponding phenotypes (IFNγ-BCG or IFNγ-ESAT6_BCG_) according to the genotypes at the associated SNPs. Using the original Illumina Linkage IVb markers, we then performed a linkage analysis on this adjusted phenotype, using the maximum likelihood binomial (MLB) model-free method^30^ with a trait distribution in deciles, as previously described^15^. We assessed whether the decrease in LOD-score observed after adjustment corresponded to a significant contribution of the variants to the linkage signals, by carrying out the same phenotype adjustment and linkage analysis on 1215 and 1109 randomly selected variants belonging to the OmniExpress beadchip, with a MAF >0.02 and a pairwise correlation coefficient r^2^ <0.5 from the same linkage regions on chromosome 3 and chromosome 8, respectively. Analyses of these variants provided empirical distributions of LOD-scores for comparison with the values obtained for the associated SNPs. We investigated the LD pattern of the most interesting associated SNPs within the five superpopulations of the 1000 genomes project Phase 3 (Europeans, East Asians, South Asians, Africans and Admixed Americans) with the LDlink web application (https://analysistools.nci.nih.gov/LDlink/)^31^.

## Results

### The IFNγ-BCG phenotype

**Figure 1:**
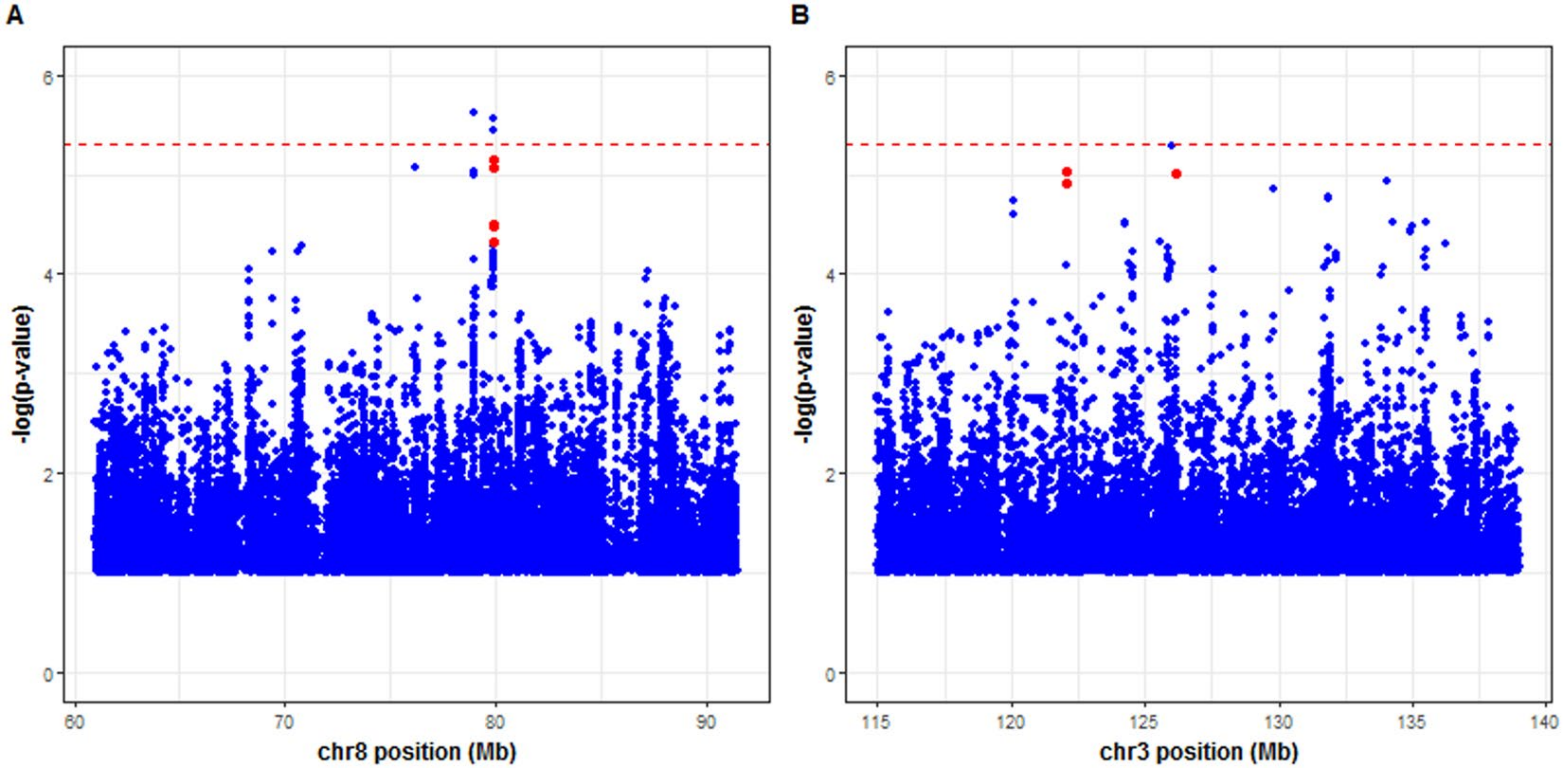
Manhattan plots displaying genetic association results for 489 related individuals from the Val-de-Marne sample using a linear mixed model approach implemented in GEMMA software (**A**) The IFNγ-BCG phenotype across 117354 SNPs in the chromosome 8 region from 61 Mb to 91.5 Mb, and (**B**) The IFNγ-ESAT6_bcg_ phenotype across 93218 SNPs in the chromosome 3 region from 115 Mb to 139 Mb. The −log 10 value of the minimum *p-*value obtained in the additive, dominant and recessive tests, is displayed against chromosomal position, in Mb, in the chromosomal region concerned. A horizontal line at a −log10 *p* value of 5 × 10^−6^ indicates the significance threshold, and points in red represent the SNPs belonging to clusters investigated in more detail after replication analyses.

We first investigated the genomic region linked to the IFNγ production in PBMCs following live BCG stimulation. In the region of interest on chromosome 8 extending from 61 Mb to 91.5 Mb, 6219 variants were genotyped in the French sample. After phasing with SHAPEIT^21^, more than 324,000 variants were imputed with IMPUTE2^23^ in this 30.5 Mb region. After quality control, we retained a total of 117,354 variants with a MAF >2% and an information criterion >0.6 for analysis with GEMMA software^24^ for association with the IFNγ-BCG phenotype (Fig. 1A). In total, 23 variants from four different LD clusters had *p*-values for association < 5 × 10^−5^. The strongest association signals were obtained with rs202163431 (*p* = 2.4 × 10^−6^, LD cluster 8-2, information criterion = 0.65), and rs6981743 (*p* = 2.7 × 10^−6^, LD cluster 8-4, information criterion = 0.98). Both these SNPs are intergenic and located more than 150 kb away from the nearest protein-coding gene (Table S1).

We carried out a replication analysis for these 23 associated SNPs in the South African sample. Only the five SNPs of cluster 8.3 met the criteria for replication. One of these five SNPs, rs12056450, was selected for genotyping, as it was also one of the most significant SNPs in the South African sample. Genotyping was successful in 368 individuals from the French sample and in 236 individuals from the South African sample. In the genotyped individuals, the concordance between the imputed genotypes and the real genotypes was 0.96 for the Val-de-Marne sample and 0.99 for the Tygerberg sample, confirming the high quality of imputation (Table S2). We therefore replaced the imputed dosage data with the real genotypes when available or with best-guess genotypes otherwise, and repeated the association analyses for this SNP. The results of the association study are shown in Table 1. With an additive model, rs12056450 SNP had a slightly lower *p*-value (1.16 × 10^−5^) in the Val-de-Marne sample, but this SNP was not significantly replicated in the Cape Town sample (*p* = 0.25) with the same genetic model. The frequency of the minor G allele ranged from 0.13 in subjects of African origin to 0.47 in Caucasians from the French sample, with an intermediate value of 0.23 for the South African sample. The effect of the SNP, with the allele G associated with high IFNγ-BCG values, displayed some heterogeneity between populations, with African GG homozygotes having a very low phenotype value in the French sample, and AG heterozygotes having slightly lower mean phenotype values than AA homozygotes in the South African sample (Figure S2).

**Table 1:** Association results for IFNγ-BCG and IFNγ-ESAT6_bcg_ phenotypes, based on genotyping data for the 3 selected variants according to the criteria described in the Methods, for the French and South African samples.

LD cluster*	Position (bp)	SNP	Alleles**	Genetic Model***	Val-de Marne Sample			Tygerberg Sample		
					AF ^**^	Estimated effect(SE)^#^	p-value	AF	Estimated effect(SE)^#^	p-value
**IFNγ**_**-**_ **BCG**										
8-3	79887368	rs12056450	G/A	Additive	0.31	0.36 (0.08)	1.2 × 10^−5^	0.23	0.06 (0.09)	0.25
**IFNγ-ESAT6**_**bcg**_										
3-2	122059775	rs9784373	T/A	Dominant	0.05	0.72 (0.17)	1.9 × 10^−5^	0.02	0.31 (0.30)	0.14
3–5	126129646	rs9828868	T/C	Recessive	0.49	0.49 (0.11)	9.6 × 10^−6^	0.46	0.12 (0.13)	0.19

*LD cluster as defined in Tables S1 and S3.

**The first mentioned allele is associated with high phenotype values and AF = allele frequency for the first allele mentioned. ***Genetic model for the allele mentioned in the Table.

^#^Estimated effect = regression coefficient with its standard error (SE).

The cluster of SNPs tagged by rs12056450 included 31 variants with r^2^ values >0.8 in the French sample. This 40 kb block is located in an intergenic region starting at the end of the LOC105375914 non-coding RNA gene, and the nearest protein-coding gene, encoding IL7, is located 140 kb away. Among the bin SNPs, rs12682556 (r^2^ = 0.97 with rs12056450 in the Val-de-Marne sample, *p*-value for association with IFNγ-BCG of 5.1 × 10^−5^) is referenced as corresponding to a 400 bp binding region of the CTCF transcription factor in the RegulomeDB database^32^. Finally, we investigated whether rs12056450 contributed to the linkage signal observed on chromosome 8 for the French sample, by adjusting the IFNγ-BCG values for the corresponding SNP. Adjustment for rs12056450 decreased the LOD score from 3.80 to 3.44. We assessed the significance of this result, by calculating an empirical distribution of LOD scores (see methods). We found that the decrease in LOD score observed with rs12056450 was not significant (empirical *p*-value of 0.14) (Figure S3A).

### The IFNγ-ESAT6_BCG_ phenotype

Next, we focused on the locus impacting the IFN-γ production after *M. tuberculosis* specific ESAT-6 stimulation adjusted for the IFN-γ amount triggered by live BCG. In total, 5901 variants were genotyped and more than 249,000 were imputed within the 24 Mb linkage region of chromosome 3, by the same strategy as used for chromosome 8. Studies of association with the IFNγ-ESAT6_BCG_ phenotype were conducted in the French sample, with 93,218 variants having a MAF >2% and an information criterion >0.6. Figure 1B shows the results for the most significant association for the three genetic models tested (additive, recessive, dominant). In total, 17 variants from nine independent LD clusters provided a *p*-value for association < 5 × 10^−5^ (Table S3). The strongest association signal was that for the imputed SNP rs116817490 (*p* = 5 × 10^−6^, information criterion = 0.82) located 14 kb downstream from the *KLF15* gene. We investigated these 17 associated SNPs in the Tygerberg sample: three, from two independent clusters, met the criteria for replication described in the methods (Table S3).

Two of these three selected variants (rs9784373 and rs149692729) had been imputed, and were found to be in strong LD. Only one of these two SNPs (rs9784373) was used in subsequent analyses, together with the independent SNP rs9828868, which had already been genotyped in the French sample. The concordance between the imputed and real genotypes was high, ranging from 0.86 to 0.99, confirming the accuracy of imputation (Table S2). We therefore replaced the imputed dosage data with the real genotypes when available or with best-guess genotypes otherwise, and repeated the association analyses for these two SNPs (Table 1). SNP rs9784373 yielded similar results with the dominant model in the French sample, but the initial evidence of association in the Tygerberg sample (*p = *1.7 × 10^−5^) became much weaker and was no longer significant after genotyping (*p = *0.14). The frequency of this variant was low (<0.04) in most populations other than the African subpopulation of the Val-de-Marne sample (MAF = 0.1), with also some heterogeneity of the genetic effect in the Caucasian subpopulation of the Val de Marne sample (Figure S4), making this association result more difficult to interpret.

**Figure 2:**
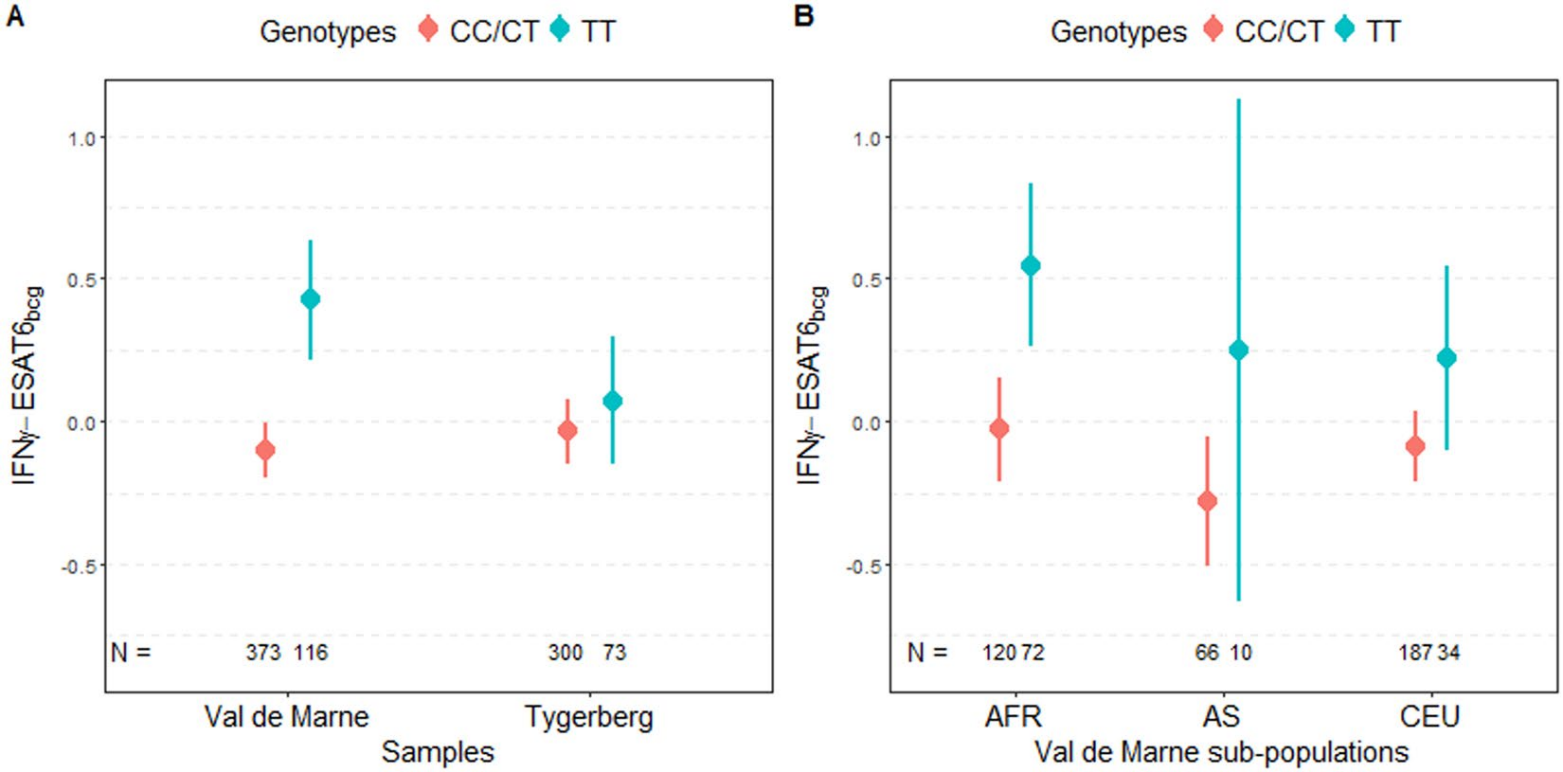
Distribution of IFNγ-ESAT6_bcg_ means by rs9828868 genotype in (**A**) Val-de-Marne and Tygerberg samples, and in (**B**) different subpopulations of the Val-de-Marne sample. The dots correspond to the means and the error bars correspond to the 95% confidence interval of the mean calculated under an assumption of normality. The IFNγ-ESAT6_bcg_ phenotype was standardized.

**Figure 3:**
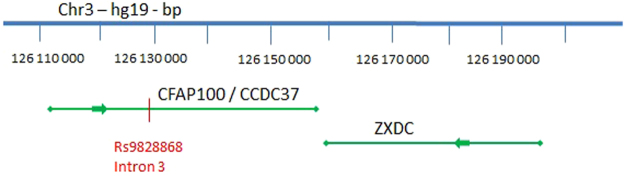
Localization of rs9828868 on chromosome 3 according to hg19 coordinates. The SNP is located within the *CFAP100* gene (cilia and flagella associated protein 100), approximately 30 kb from its target gene *ZXDC* (zinc finger X-linked duplicated family member C).

SNP rs9828868, which had already been genotyped in the French sample (association *p*-value of 9.6 × 10^−6^ under a recessive model), displayed a slight improvement in its *p*-value for association after genotyping in the Tygerberg sample, from 0.22 to 0.19 under the same recessive model (Table 1). Its minor allele T had a frequency of 0.49 in the French sample and 0.46 in the Tygerberg sample. Homozygous TT individuals had higher IFNγ-ESAT6_bcg_ values than CC and CT individuals (difference of ~0.5 standard deviations in the French sample) (Fig. 2A). This effect was homogeneous in the three main populations (Caucasian, African, and Asian) of the French sample (Fig. 2B). Overall, this SNP accounted for 15% of the genetic variance of the distribution of the IFNγ-ESAT6_bcg_ phenotype in the French sample. We investigated the LD pattern of rs9828868 in the populations of the 1000 Genomes project Phase 3. We found only one SNP with an r^2^ >0.8, rs4679239, in European and Asian superpopulations. This SNP was imputed, and was not strongly associated with the IFNγ-ESAT6_bcg_ phenotype in the French sample. This association therefore appears to be driven by a single SNP, rs9828868, located in intron of the *CFAP100* gene (cilia and flagella associated protein 100 or *CCDC37*) (Fig. 3).

Finally, we investigated whether these two putative associated variants could account, at least in part, for the chromosome 3 linkage signal, by adjusting the IFNγ-ESAT6_bcg_ values for the corresponding SNPs. Following the same strategy as for the IFNγ-BCG phenotype, we computed an empirical distribution of LOD scores (see methods). After adjustment for rs9784373, the LOD score in the French sample was 3.05, close to the initial value of 3.26 obtained with the same individuals (empirical *p*-value of 0.22). By contrast, when we adjusted for rs9828868, the LOD score decreased from 3.26 to 2.05. This fall in LOD score was highly significant (empirical *p*-value < 10^−3^), and was larger than those obtained with 1215 randomly chosen independent variants (Figure S3B). Overall, our analyses of the chromosome 3 region identified rs9828868 as the SNP for which the evidence for association with the IFNγ-ESAT6_bcg_ phenotype was the strongest. Interestingly, rs9828868 has also been reported to be a significant expression quantitative trait locus (eQTL) associated with expression of the nearby *ZXDC* (zinc finger X-linked duplicated family member C) gene in whole blood cells^33^.

## Discussion

In this study, we conducted fine mapping of two previously described linkage loci^15^ through high-density genotyping and imputation. For the 8q12-22 locus controlling the production of IFN-γ by PBMCs following stimulation with live BCG, we identified, in the French sample, a suggestive association with a cluster of intergenic SNPs tagged by rs12056450. This cluster presented the same trend of association in the Tygerberg sample, with the same genetic model as in the Val-de-Marne. However, the effect on IFN-γ levels observed in AG heterozygotes in the French sample was not observed in AG heterozygotes from the South African sample. This cluster of SNPs did not explain a substantial part of the linkage signal for chromosome 8, and further studies are required to confirm or rule out a role for this cluster in IFN-γ production in response to BCG stimulation.

The second major locus on chromosome 3q13-22 was found to control the IFN-γ production induced by ESAT-6 antigen after taking into account the amount shared with that induced by BCG. Our analyses identified a single common C/T variant, rs9828868, with a *p*-value of 9.6 × 10^−6^ in the French sample and a trend for association, under the same genetic model, in the Tygerberg sample. This weaker association in the Tygerberg sample was not unexpected given the weaker linkage signal obtained in the primary study. Subjects homozygous for the T allele had higher values than individuals with C/T and CC genotypes, with a difference of ~0.5 SD in the French sample, accounting for 15% of the genetic variance of the IFNγ-ESAT6_BCG_ phenotype. This effect was homogeneous across the three main subpopulations of the Val-de-Marne sample (Caucasians, Africans, and Asians) (Fig. 2B). This variant also made a significant contribution to the linkage signal on chromosome 3, providing strong evidence for a genuine association.

SNP rs9828868 was reported to be an eQTL of the *ZXDC* gene in blood cells, with the T allele of the variant being associated with low levels of *ZXDC* expression^33^. The product of *ZXDC* was first described as a zinc finger protein that binds CIITA and contributes to the transcription of MHC class II genes^34^. It also regulates the expression of genes involved in monocyte differentiation and function. In particular, the largest isoform, ZXDC1, activates the expression of *CCL2* (chemokine ligand 2, also known as monocyte chemoattractant protein 1, MCP-1) by evicting the transcriptional repressor BCL6^35^. *ZXDC* knockdown leads to an increase in the occupancy of the *CCL2* promoter by BCL6 following PMA induction, and to lower levels of *CCL2* expression. Individuals carrying the T allele of rs9828868, and TT homozygotes in particular, may have lower levels of *ZXDC* expression, resulting in lower levels of CCL2 induction. Several studies of human cells *in vitro* studies have reported that CCL2 inhibits IL-12 production^36^, particularly in *M. tuberculosis*-stimulated monocytes^37^. All these observations are consistent with the view that TT homozygotes have higher IFNγ-ESAT6_BCG_ levels due to an increase in IL-12 production triggered by the *ZXDC*-dependent downregulation of CCL2.

In conclusion, we identified rs9828868 as associated with the production of IFN-γ by PBMCs following stimulation with the ESAT-6 antigen, in an ethnically heterogeneous sample from Val-de-Marne, after adjustment for the levels of IFN-γ production common to BCG and ESAT-6 stimulation. This common element may reflect a general capacity for IFN-γ production via the TCR signaling pathway, whereas the IFNγ-ESAT6_BCG_ phenotype is thought to be more specific to ESAT-6, and, consequently, to *M. tuberculosis*, as this antigen is absent from the BCG strain. This variant explains a significant part of the linkage peak previously identified for the same sample, and is involved in expression of the *ZXDC* gene, which is biologically linked to IL-12 production. In the Tygerberg replication sample, the same allele was associated with high values of the studied trait, although this association with not significant at the 5% level. The phenotypes used for replication in South Africa were similar, but not identical to those used in the French sample (IFN-γ production in whole-blood samples vs. PBMCs, measured at 3 days vs. 4 days, respectively), and these slight differences may have led to a loss of replication power.

Moreover, the two populations differed considerably in terms of their exposure to *M. tuberculosis*. The studied individuals from South Africa live in an area of hyperendemic tuberculosis, in which *M. tuberculosis* transmission occurs preferentially in the community^38^. By contrast, tuberculosis endemicity is low in France, and the design of the French study targeted household tuberculosis contacts. The two cohorts also differed in terms of genetic background. The families included in the French sample belonged to several different ethnic groups, whereas all the individuals from the replication sample studied were from the South African Coloured ethnic group, a population resulting from an admixture of Khoesans (31%), Bantu-speaking Africans (33%), Europeans (16%), and Asians (20%)^39^. In this context, the result obtained for rs9828868, which seems to be robust to ethnic and environmental heterogeneity, is particularly promising, and provides new clues to the mechanisms of anti-tuberculosis immunity in humans.

## Electronic supplementary material

Supplementary Data

Supplementary Tables
