# Supplementary material for: An eQTL variant of ZXDC is associated with IFN-γ production following Mycobacterium tuberculosis antigen-specific stimulation
Source: Sci Rep. 2017 Oct 9;7:12800. doi: 10.1038/s41598-017-13017-8 (PMC5634485; doi:10.1038/s41598-017-13017-8)
Supplement: Supplementary file 1 — Supplementary Data [file 41598_2017_13017_MOESM1_ESM.doc]

**An eQTL variant of *ZXDC* is associated with IFN-γ production following**

***Mycobacterium tuberculosis* antigen-specific stimulation**

Fabienne Jabot-Hanin^1,2^, Aurélie Cobat^1,2^, Jacqueline Feinberg^1,2^, Marianna Orlova^3,4^, Jonathan Niay^5^, Caroline Deswarte^1,2^, Christine Poirier^6^, Ioannis Theodorou^5^, Jacinta Bustamante^1,2^, Stéphanie Boisson-Dupuis^1,2,7^, Jean-Laurent Casanova^1,2,7,10,11^, Alexandre Alcaïs^1,2^, Eileen G. Hoal^8^, Christophe Delacourt^9^, Erwin Schurr^3,4^, and Laurent Abel*^1,2^,

**Supplementary Data**

**Methods - Subjects and families from Val de Marne**

From April 2004 to January 2009, household contacts exposed to a patient with culture confirmed pulmonary tuberculosis (TB) were enrolled in the context of a general screening procedure in Val de Marne, suburb of Paris. In France, all new cases of TB are reported to the health authorities (Centre de Lutte contre la Tuberculose) to organize epidemiologic investigations and to identify contact subjects with the consent of the index case. A household contact was defined as any person sharing the residence of a TB index case during the three months preceding diagnosis of the case. A questionnaire was completed for each individual to assess the risk factors for infection and the familial relationships as detailed in (1,2).

**Methods - Significance thresholds and inflation factors**

For each of the two studied phenotypes, we computed the genomic inflation factor λ on imputed variants with a MAF > 2% and pruned on the basis of r^2^ < 0.1 using the GenABEL package(6). For the IFNγ-BCG phenotype analysis, we did not use variants located on the chromosome 8, and found a λ = 1.032. For the IFNγ-ESAT6_BCG_ phenotype analysis, we removed variants located on chromosome 3, and found a λ = 1.025. We also computed a precise region-wide significance threshold based on the effective number of independent markers in each region to check if our estimation of 5.10^-6^ was a reasonable significance threshold for each of the 2 regions. For that, we thinned the imputed markers with a MAF > 2% thanks to PLINK software (3,4) using a window of 5000 SNPs, a shift of 5 SNPs and a r^2^ < 0.2, as suggested in (5). The thresholds thus calculated were of 1.15 10^-5^ for the chromosome 8 region and of 1.3 10^-5^ for the chromosome 3 region. When these thresholds were corrected by the corresponding genomic inflation factor, they became 8.3 10^-6^ and 1.0 10^-5^ for chromosomes 8 and 3, respectively. These thresholds were therefore close to our approximated thresholds of 5.10^-6^ which appeared quite reasonable and appropriate.

References

1. Aissa K, Madhi F, Ronsin N, Delarocque F, Lecuyer A, Decludt B, et al. Evaluation of a Model for Efficient Screening of Tuberculosis Contact Subjects. Am J Respir Crit Care Med. 2008 May;177(9):1041–7.

2. Cobat A, Poirier C, Hoal E, Boland-Auge A, Rocque F de L, Corrard F, et al. Tuberculin Skin Test Negativity Is Under Tight Genetic Control of Chromosomal Region 11p14-15 in Settings With Different Tuberculosis Endemicities. J Infect Dis. 2015 Jan 15;211(2):317–21.

3. Purcell S, Neale B, Todd-Brown K, Thomas L, Ferreira MAR, Bender D, et al. PLINK: a tool set for whole-genome association and population-based linkage analyses. Am J Hum Genet. 2007 Sep;81(3):559–75.

4. Chang CC, Chow CC, Tellier LC, Vattikuti S, Purcell SM, Lee JJ. Second-generation PLINK: rising to the challenge of larger and richer datasets. GigaScience. 2015;4:7.

5. Sobota RS, Shriner D, Kodaman N, Goodloe R, Zheng W, Gao Y-T, et al. Addressing Population-Specific Multiple Testing Burdens in Genetic Association Studies. Ann Hum Genet. 2015 Mar 1;79(2):136–47.

6. Karssen LC, van Duijn CM, Aulchenko YS. The GenABEL Project for statistical genomics. F1000Research. 2016 May 19;5:914.

**Supplementary Figures**

Figure S1: Distributions of adjusted standardized phenotypes IFNγ-BCG (A) and IFNγ-ESAT6_bcg_ (B) used for association analyses in the Val-de-Marne sample.


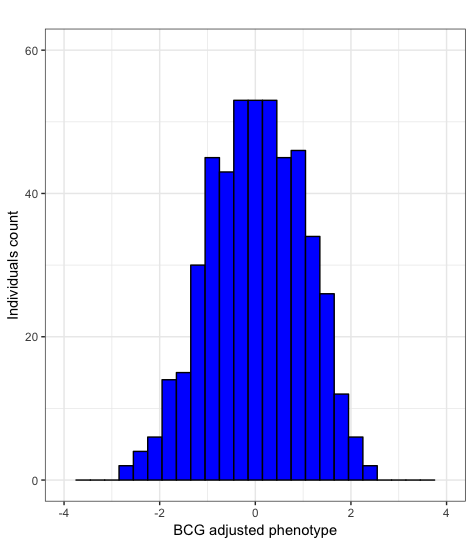


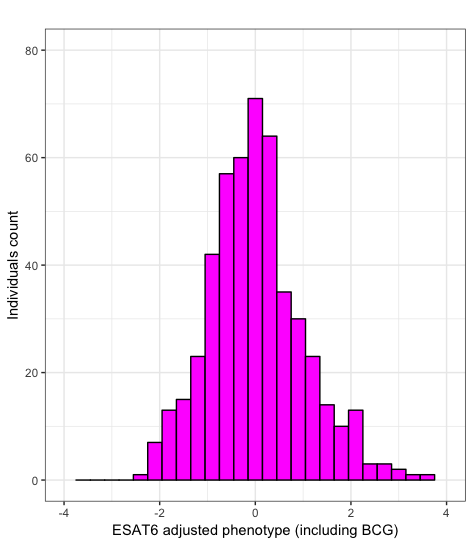
A B

Figure S2: Distribution of the IFNγ-BCG means according to rs12056450 genotypes in Val-de-Marne sample and in Tygerberg sample (**A**) and in the different sub-populations of the Val de Marne sample **(B).** The dots correspond to the means and the error bar to the 95% confidence interval of the mean computed under the normality assumption. IFNγ-BCG phenotype is standardized.


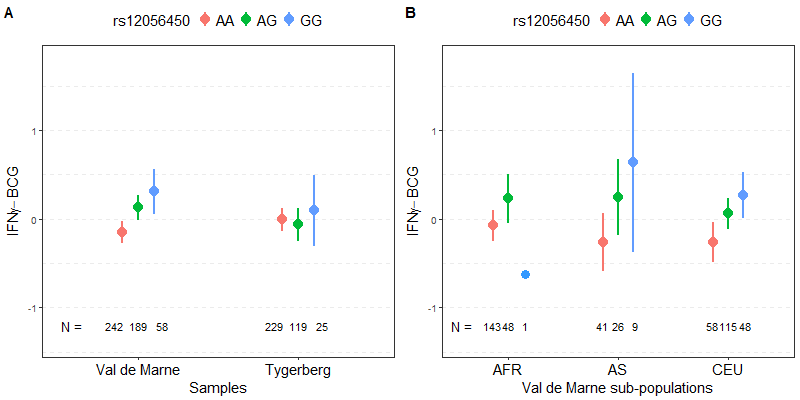


Figure S3: Empirical LOD score distributions used for assessing the contribution of the associated SNPs to the linkage peak. Figure shows the distribution of LOD scores obtained after adjustment of IFNγ-BCG phenotype on 1109 randomly selected imputed variants belonging the chromosome 8 linkage region (115 – 139 Mb) **(A)** and adjustment of IFNγ-ESAT6_bcg_ phenotype on 1215 randomly selected imputed variants belonging the chromosome 3 linkage region (115 – 139 Mb) **(B)**, all with a MAF > 2% and an info criteria >0.6. * corresponds to the LOD score obtained after adjustment on rs12056450 **(A)** and rs9828868 **(B)**. corresponds to the LOD score obtained after adjustment on rs9784373 **(B).**


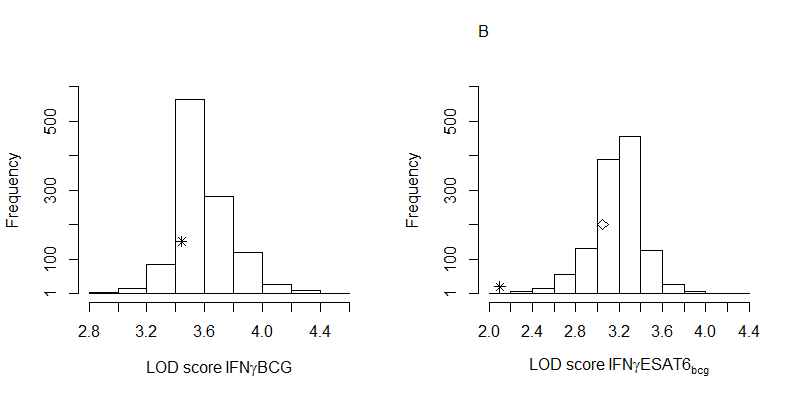


Figure S4: Distribution of the IFNγ-ESAT6_BCG_ means according to rs9784373 genotypes in Val-de-Marne sample and in Tygerberg sample (**A**) and in the different sub-populations of the Val de Marne sample **(B).** The dots correspond to the means and the error bar to the 95% confidence interval of the mean computed under the normality assumption. IFNγ-ESAT6_bcg_ phenotype is standardized.


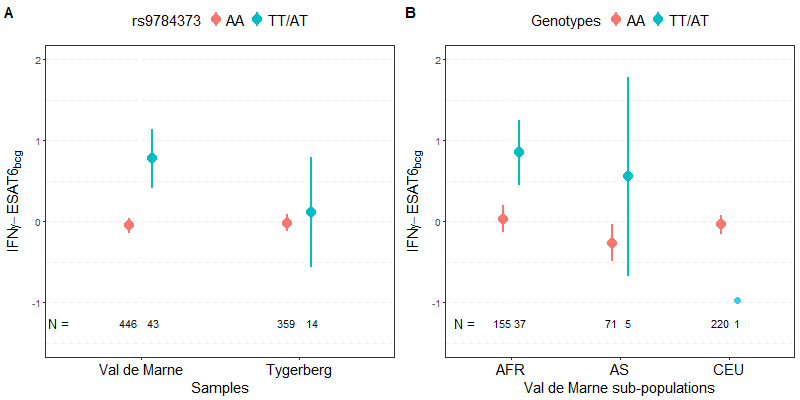


**Supplementary Tables**

Table S1: Association results on imputed data for IFNγ-BCG phenotype for p-value < 5.10^-5^ in Val-de-Marne sample

Table S2: Comparison of the bestguess genotypes coming from IMPUTE2 software versus the real genotypes on 368 individuals from Val-de-Marne and 236 individuals from Tygerberg sample

Table S3: Association results on imputed data for IFNγ-ESAT6_bcg_ phenotype for p-value < 5.10^-5^ in Val-de-Marne sample
